# Cerebro-Spinal Fever

**Published:** 1872-08

**Authors:** Charles P. Gordon

**Affiliations:** Dalton, Georgia


### CEREBRO-SPINAL FEVER.

BY CHARLES P. GORDON, M.D., DALTON, GEORGIA.

From the year 1806, when cerebro-spinal fever first made its advent to this continent, down to the present time, various regions of our country have been visited by epidemics of this fearful malady: a disease fearful from the suddenness of its onset and uniform fatality—fearful from the fact that hitherto it has baffled the skill and mocked the earnest efforts of our noble and learned profession. Unlike other diseases, whose approach its more gradual, its attack is at once upon the very citadel of human life—damming up the life-current at its source, and commencing, where almost every other fatal disease ends, in the death of its unfortunate victim. Amidst the tender and helpless years of childhood and early adolescence, it makes its greatest ravages and prefers its dreaded carnival. And must we still fold our arms, and quietly observe the dreadful scene, without a remedy to stay its progress?

We have no very definite knowledge of the causation of cerebro-spinal fever. Like the wind, "it bloweth where it listeth; we know not whence it cometh, nor whither it goeth." Reasoning from its baneful effects as expressed in disease, we would conclude that, like typhoid fever, typhoid pneumonia, and other kindred diseases, it is a systemic malady, depending upon a specific cause, with a local manifestation, involving the cerebro-spinal axis in congestion and inflammation. As in typhoid fever, the affection of the Peyerian or agminated and solitary glands of the small intestines is a local expression of a systemic malady; and as in pneumonia typhoides, the pleuro-pulmonic congestion and inflammation are secondary and consequential, and the result of a morbific agent operating through the general system, and manifesting itself locally in the involvement of those organs; so cerebro-spinal fever is a constitutional disease, with its peculiar local tendency to the nervous centres. This, I believe, is its recognized pathology. What, then, would be a rational conclusion, deduced from this view of its pathology, as to its treatment? That we should carefully husband the resources of the system, and not, with a dauntless heroism, pour out the life-blood of the patient, until it can scarcely ebb and flow; not forgetting, that should the patient withstand the first onset of the disease, and recover partially from its withering assault, his system will be taxed to its utmost, requiring all its vital energy to carry the patient safely through the disease; remembering that cerebro-spinal fever, like typhoid fever and typhoid pneumonia, is an adynamic disease, protracted frequently to an indefinite extent, prostrating the vital forces, and tending to death by asthenia.

A case in point came under my observation at this place, which I will briefly mention, as aiding to illustrate my subject and establish the thesis I am endeavoring to maintain.

A colored man was found in a state of profound coma and insensibility, with tonic contraction of cervical and dorsal muscles, trismus, and opisthotonos—which was diagnosed, cerebro-spinal fever. The patient, being unable to swallow, was subjected to the inhalation of chloroform, and afterward to purgation, with counter-irritation to the spine, when he gradually recovered consciousness, with partial relaxation of muscular contraction, cervical muscles remaining in a state of tonic contraction, with low febrile reaction and delirium. The case assuming in its progress a typhoid type, a supporting treatment was adopted, with alcoholic stimulants in moderation, and the oil of turpentine. The case progressed favorably until the end of the fourth week, when the patient was considered convalescent, and discharged. From an indiscretion in diet several days afterward, we were informed, he relapsed and died.

The patient in this case, after recovering from the first violence of the disease, passed through a long and tedious attack of four weeks' duration, which taxed his constitutional vigor to its utmost resource, and under a supporting treatment, would have entirely recovered but for his own indiscretion.

This case, with others I have observed, attests the wisdom of not recklessly sacrificing the strength of the patient in the early stages, by blood-letting and other remedies, permanently lowering the vital powers.

In cases of cerebro-spinal fever in which the nervous centres are overwhelmed with congestion and inflammation by the intensity of the materies morbi, producing coma and insensibility, with tonic muscular spasm, rendering deglutition impossible, and the administration of medicines per ore impracticable, I would resort to the inhalation of chloroform to relieve cerebral congestion and relax muscular contraction, with stimulating pediluvia, and severe counter-irritation to the spine, as balancive measures tending to equalize the circulation. The violence of the disease abating, or in cases not attended with such initial violence, I would administer large doses of the bromide of potassium during the day, and quiet the patient at night with the hydrate of chloral, which, being a recognized cerebral sedative, I should regard preferable to any form of opium. In the latter stages, the patient should be supported with good nourishment and alcoholic stimulants. I would not administer the sulphate of quinine, unless in regions of country where the disease might be modified by malarial influence coöperating with its peculiar cause, or in the latter stages, when it might be prescribed as a tonic. Opium I would discard, especially in the earlier stages, as tending to increase cerebral congestion. The oil of turpentine I would use in the latter stages, as an arterial stimulant and eliminative remedy; while the tincture of gelseminum sempervirens may be advantageously combined with the bromide of potassium.

These crude remarks are respectfully submitted, and if deemed worthy of a place in the JOURNAL, are at your service.

